# A novel task to evaluate irony comprehension and its essential elements in Spanish speakers

**DOI:** 10.3389/fpsyg.2022.963666

**Published:** 2022-11-22

**Authors:** Elizabeth Valles-Capetillo, Cristian Ibarra, Domingo Martinez, Magda Giordano

**Affiliations:** ^1^Instituto de Neurobiología, Universidad Nacional Autónoma de México, Juriquilla, Mexico; ^2^Escuela Nacional de Estudios Superiores, Universidad Nacional Autónoma de México, Querétaro, Mexico

**Keywords:** irony, contextual discrepancy, prosody, facial expression, theory of mind

## Abstract

An ironic statement transmits the opposite meaning to its literal counterpart and is one of the most complex communicative acts. Thus, it has been proposed to be a good indicator of social communication ability. Prosody and facial expression are two crucial paralinguistic cues that can facilitate the understanding of ironic statements. The primary aim of this study was to create and evaluate a task of irony identification that could be used in neuroimaging studies. We independently evaluated three cues, contextual discrepancy, prosody and facial expression, and selected the best cue that would lead participants in fMRI studies to identify a stimulus as ironic in a reliable way. This process included the design, selection, and comparison of the three cues, all of which have been previously associated with irony detection. The secondary aim was to correlate irony comprehension with specific cognitive functions. Results showed that psycholinguistic properties could differentiate irony from other communicative acts. The contextual discrepancy, prosody, and facial expression were relevant cues that helped detect ironic statements; with contextual discrepancy being the cue that produced the highest classification accuracy and classification time. This task can be used successfully to test irony comprehension in Spanish speakers using the cue of interest. The correlation of irony comprehension with cognitive functions did not yield consistent results. A more heterogeneous sample of participants and a broader battery of tests may be needed to find reliable cognitive correlates of irony comprehension.

## Introduction

Pragmatics studies the role that language plays in social communication and how contextual elements can facilitate this process. Pragmatic abilities have been described as the proficiency to communicate, express, and recognize intentions (Scott-Phillips, 2017). They represent a key process in human communication, allowing people to distinguish between the possible alternative interpretations of the linguistic information they receive (Bosco et al., 2017). Alteration in social communication has been reported in several disorders, for example: the Social Communication Disorder and the Autism Spectrum Disorder (American Psychiatric Association, 2013). One of the most difficult communication forms to understand is irony (Wilson and Sperber, 1981), therefore it has been proposed that it can be a useful indicator of pragmatic abilities (Caillies et al., 2014). Irony plays different roles during communication; it serves to indirectly convey feelings (Shamay-Tsoory et al., 2005), express courtesy, emotion, or humor, and enhance criticism (Milanowicz, 2013). It has been reported that ironic statements are used in approximately 7% of the conversational turns in everyday conversation (Tannen, 2005), and 8% during conversations with friends (Gibbs, 2000).

One of the most utilized theories to understand irony is the standard pragmatic view (Grice, 1975), which proposes that when an ironic statement is comprehended, the receiver or listener of the message first constructs the literal interpretation, and when it becomes apparent that the literal interpretation is not compatible with the context, the ironic interpretation is established. From this view, ironic interpretation requires more effort, resources, and time from the listener. In opposition with Grice, Gibbs (1994) proposed the direct-access view theory. This theory assumes that the contextual and lexical information is processed interactively in early stages, and if context supports an ironic interpretation, this can be activated directly, without the need for the literal interpretation to be computed first (Gibbs, 1994). Compared with the standard pragmatic view, the direct-access view suggests that irony does not require more time from the receptor. Likewise, the graded salience hypothesis states that salient meanings are activated initially, giving a limited role to context. Giora defined salience as “the accessibility of meanings of words or collocations out of context.” If there are salient cues that support the ironic interpretation, it would be computed first (Giora, 1997).

In addition to the above theories, Attardo (2000) proposed that certain psycholinguistic properties are important for the identification of ironic statements. One of them is the relevance that a statement has to its context. Another is the appropriateness of a statement to its context, which indicates whether the linguistic information of the statement is compatible with the information available in the context. A third property is the speaker’s intention. In the case of ironic statements, the intention is that the listener detects the true message (i.e., ironic). According to this view, ironic statements are relevant, inappropriate to the context, and are used by the speaker to convey the true meaning to the listener (Attardo, 2000).

Pexman (2008) proposed the constraint satisfaction model for the processing of ironic statements. According to this model, cues activated by a statement “are processed rapidly and in parallel and an ironic interpretation is considered as soon as there is sufficient evidence that it might be supported “(Pexman, 2008, p. 287). The correct selection of the intended meaning depends on the adequate functioning of the speech recognition system, and on the cues that are activated by the statement including event comprehension (outcome and history), statement valence, the frequency of irony usage in a situation, the speaker’s attitude (e.g., facial expression and prosody), and the listener’s expectations. These elements are supported by the Theory of Mind (ToM), executive functions, and the listener’s experience with irony (Pexman, 2008).

The ToM is the ability to represent mental states of oneself and others, such as desires, beliefs, emotions, and intentions (Premack and Woodruff, 1978). Because the linguistic code may not be enough to represent the full meaning of language during social communication, ToM plays an important role filling this gap (Bohrn et al., 2012; Spotorno et al., 2012). Executive functions include the ability to inhibit unwanted behaviors, to update information or strategies to solve problems (Miyake et al., 2000). Executive functions measures can predict the pragmatic performance in patients with brain injury (Bosco et al., 2017), thus it has been proposed that these functions are relevant for pragmatic comprehension⁠. In older adults it has been reported that the identification of irony has an association with inhibitory control, mental flexibility and working memory (Gaudreau et al., 2015).

With regard to the cues for the identification of irony, the discrepancy between the context and the statement is considered a relevant cue (Kreuz and Link, 2002). Other cues that can facilitate the identification of irony are prosody (Wang et al., 2006) and facial expression (Akimoto et al., 2014). The acoustic parameters associated with prosody in irony are lower fundamental frequency (F0; Rockwell, 2001; Peters et al., 2016), changes in F0 (Milosky and Ford, 1997; Cheang and Pell, 2009; Bryant, 2010; Li et al., 2013; Deliens et al., 2017; Rivière et al., 2018), greater intensity (Rockwell, 2001; Li et al., 2013; Peters et al., 2016; Deliens et al., 2017), and slower speech rate (Rockwell, 2001; Cheang and Pell, 2009; Bryant, 2010; Li et al., 2013; Peters et al., 2016; Voyer and Vu, 2016; Deliens et al., 2017). The facial information that supports ironic comprehension includes smiling, raised eyebrows, eye-rolling, winking, and squinting eyes (Rockwell, 2001; Attardo et al., 2003; Caucci and Kreuz, 2012).

The neural correlate of irony comprehension has been studied using different psychophysiological tools such as electrophysiological (EEG) recordings, eye-tracking and functional magnetic resonance imaging (fMRI) (see Fabry, 2021). The literature on neuroimaging (fMRI) of irony comprehension is relatively modest, only 12 studies have been published since 2004, and none have used Spanish as the natural language (see review by Reyes-Aguilar et al., 2018). The tasks that have been used involve mostly written scenarios followed by an ironic or non-ironic utterance which the participants are asked to judge. The results of a meta-analysis of these studies showed that understanding irony requires the left language network and areas that participate in ToM (Reyes-Aguilar et al., 2018). Furthermore, the results of this meta-analysis suggested that the natural language employed may be relevant for pragmatic language processing (Reyes-Aguilar et al., 2018).

With these antecedents in mind, we aimed to create a task that evaluated the identification of ironic statements in Mexican adults that could be used for subsequent fMRI experiments. We used three cues: contextual discrepancy, prosody and facial expression. To select the cue that would lead participants in fMRI studies to identify a stimulus as ironic in a reliable way, the three cues were evaluated independently. First, we created the statements, i.e., ironic, literal, unrelated and white lies, and their accompanying contexts. Second, we assessed the psycholinguistic properties of the statements which included comprehensibility, relevance, appropriateness, sincerity, and emotional valence; all according to the context in which they were used. We also evaluated if the contexts were comprehensible. Third, we selected acoustic parameters and facial expressions indicative of irony, and evaluated if they were correctly identified. Fourth, we compared contextual discrepancy, prosody and facial expression in terms of the classification accuracy and classification time of ironic statements. Finally, to assess the relationship between ironic statement identification and cognition we applied a battery of psychometric tests that evaluate cognitive processes that have been associated with irony identification.

## Materials and methods

### Construction of linguistic stimuli

#### Contextual discrepancy

For the ironic identification task, 56 social contexts, and 14 statements were created. Each statement was associated with four different categories of social contexts. Each category of context creates an environment that modifies the interpretation of the statements (e.g., ironic). In each context, two adults of the opposite sex and the same social standing (e.g., colleagues, classmates) interact, and one of them utters the statement. The stimuli were created in Spanish, the context was 30 to 40 words long, statements were 3 to 6 words long. The operational definitions for each category of statement are as follows:

- Ironic: a statement that is relevant, meaning it has relation to the context. The information presented in the context differs from the message conveyed in the statement. The speaker intends the statement to be interpreted as ironic, i.e., to convey irony.
- Literal: a statement that is relevant, and appropriate, meaning the information presented in the context is compatible with the message conveyed in the statement. The speaker intends the statement to be interpreted literally.
- Unrelated: a statement that has no relation to the context. The information in the context disagrees with the message conveyed in the statement. There is no intention on the part of the speaker.
- White lies: a statement that is relevant, meaning it has relation to the context. The information presented in the context differs from the message conveyed in the statement. The speaker has the intention to hide the truth.

The following is an example of the target statement: “You are playing very well.” The context used to turn it into an ironic statement was: “Paco is playing soccer and Karla is watching him. Paco is playing terribly and scores an own goal. They both believe that Paco is obviously playing badly. At halftime, Paco approaches Karla. Karla tells him: *You are playing very well*.” The context used to turn it into a literal statement was: “Omar is playing cards with Lluvia. Lluvia has won almost every game. Lluvia is very cheerful because she is winning. Omar thinks that Lluvia is playing very well. Omar tells Lluvia: *You are playing very well*.” The context used to turn it into an unrelated statement was: “Verónica and Saúl are at a piano recital. The presentation is flawless and moving. Both are satisfied with the presentation. Saúl asks Verónica what she thinks of the recital. Veronica responds: *You are playing very well*.” And for white lies, the context was: “Paulina is teaching Marcos chess. Marcos makes bad moves and is losing. Paulina sees Marcos excited and does not want to discourage him. Marcos asks her how he’s playing. Paulina answers: *You are playing very well*.”

Because we used the same statements for all four context categories, only the word length of the contexts was analyzed; a one-way ANOVA showed no significant effect of context category on word length.

##### Materials and procedure

**Figure 1:**
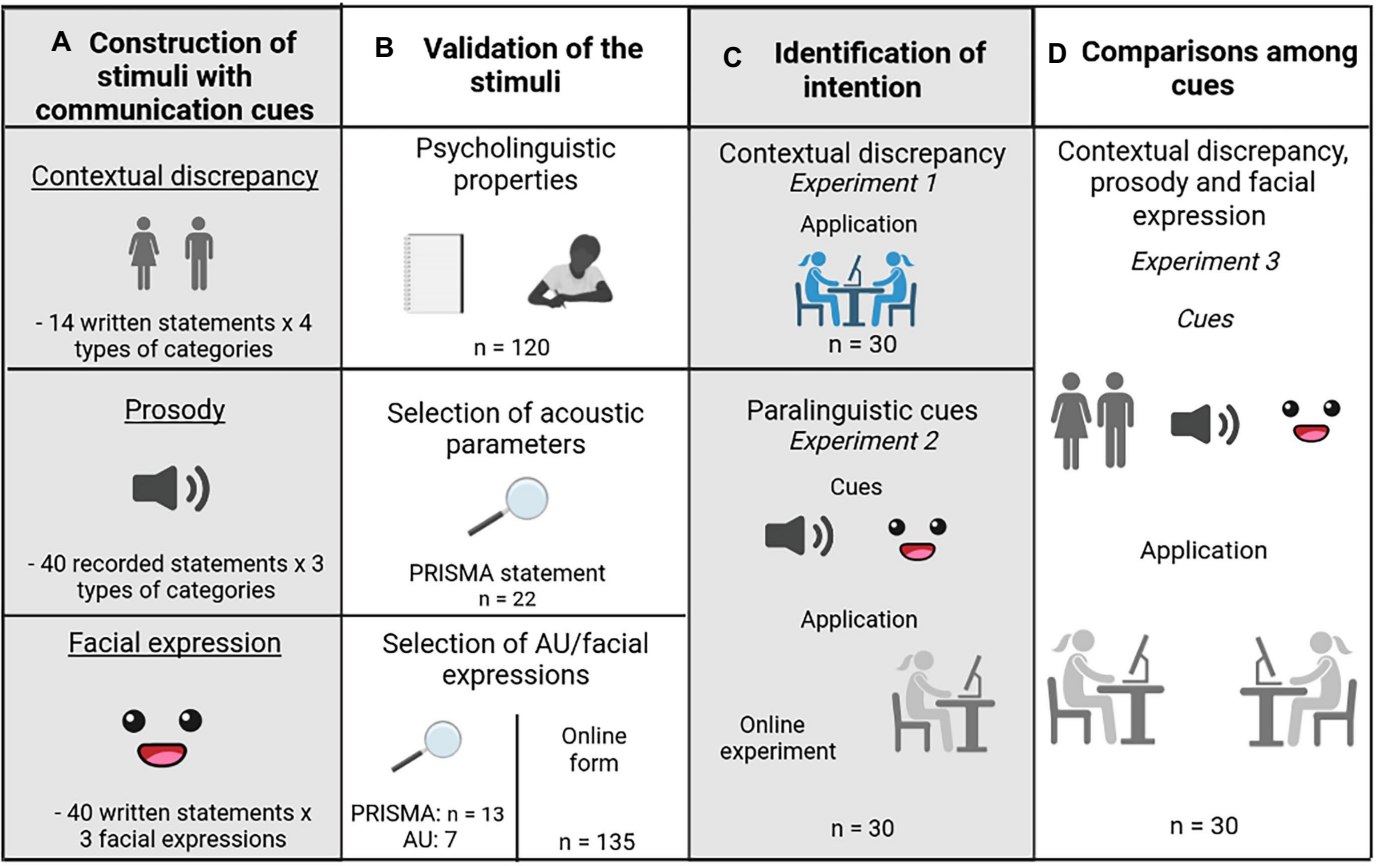
Graphical depiction of the experimental procedure. Columns show the phases of each experiment and rows depict the cues that were evaluated (i.e., contextual discrepancy, prosody and facial expression). Details about each step are available in their respective sections. AU = Action Units.

The next step was to validate if the stimuli were consistent with the psycholinguistic properties that were expected and to assess if the stimuli were accurately detected. The psycholinguistic properties evaluated were the comprehensibility of the context (without considering the statement); relevance, if the statement had relation to the context; sincerity, if the speaker wanted the listener to know the truth; appropriateness, if the statement was congruent with the contextual information; and emotional valence, if the statement, when read in a particular context, evoked a positive or negative feeling. Also, participants were asked to classify the intention of the statement according to the context (i.e., ironic, literal, unrelated, or white lies; see Figure 1).

The stimuli were organized into three booklets, each one evaluated by a separate sample of 30 participants. The psycholinguistic properties were ranked on a Likert scale of 1 to 4 points. To encourage scores to be assigned carefully, some properties ranged from higher to lower (i.e., 1 = higher comprehensibility, appropriateness, and emotional valence) and others from lower to higher (i.e., 1 = lower relevance and sincerity). The intention was classified by selecting among the four categories of statements (i.e., ironic, literal, unrelated, or white lie). Participants were asked to read the definitions of the statements that were on the first page of the booklets (for definitions of statements categories, see previous section). According to the results, 14 contexts were not understandable and had to be modified to improve their comprehensibility. The 14 modified contexts were evaluated by a different sample of 30 participants using a fourth booklet. Then, an independent sample of participants ranked how ironic they considered the ironic statements using a Likert scale of 7 points (1 = less ironic, 7 = more ironic).

##### Participants

Participants were asked to sign an informed consent form to participate in the study and to fill in a general data form with information about their level of education, sex, and age. Participants were undergraduate or graduate Spanish-speaking students that reported no psychiatric or neurological disorders. Considering the four booklets, the stimuli were evaluated by 120 participants, with a mean age of 22.91 ± 3.82 (booklet 1 = 22 F, 8 M, mean age 22.06 ± 3.34; booklet 2 = 20 F, 10 M, mean age 21.57 ± 2.57; booklet 3 = 21 F, 9 M, mean age 23.03 ± 4.00; and booklet 4 = 20 F, 10 M, mean age 23.86 ± 4.63). An additional sample of 45 participants (22 Female, mean age of 26.69 ± 5.83) ranked the 14 ironic statements, in terms of how ironic they found them.

##### Statistical analysis

For aesthetic reasons and ease of reading, results for all psycholinguistic properties were homogenized. Thus, the scores for comprehensibility, appropriateness, and emotional valence were inverted (i.e., from 1 = higher to 1 = lower). The statistical analyses were performed using R software (version 3.6.3; R Core Team, 2020) through the graphical interface of RStudio (version 1.1.447; RStudio Team, 2019). First, the descriptive statistics of classification accuracy and psycholinguistic properties were computed. The percentage and standard deviation are presented for the classification accuracy; the median (Mdn) and the interquartile range (IQR) are reported for the psycholinguistic properties.

Additionally, to analyze if the identification of statement categories could be predicted by scores of relevance, appropriateness, and sincerity, a multinomial logistic regression, was calculated and a model was designed (multinom function from the nnet package; version 7.3–17, Venables and Ripley, 2002). According to the recommendations to perform this analysis (Venables and Ripley, 2002), the data was split into two datasets, the first one was used to train the model (80% of data), and the second to validate the model (20% of data). The model was calculated four times. First, with all the statements of the four categories. Then, considering the ratings of how ironic the statements were rated, they were split into two categories: less ironic (statements: 1, 3, 5, 36, 44, 53, 55, Mdn = 6) and more ironic (statements: 10, 52, 15, 17, 22, 46, Mdn = 7). Considering these two categories (i.e., less and more ironic), the model was calculated excluding the more ironic statements; then, excluding the less ironic statements. For each category (i.e., less or more ironic) a Monte Carlo simulation, with 5,000 replications of the model, were calculated and the mean accuracy of those simulations are reported. Finally, excluding six ironic statements randomly (i.e., regardless if they were less or more ironic) 5,000 replications of Monte Carlo simulation of the model were calculated.

##### Results

**Figure 2:**
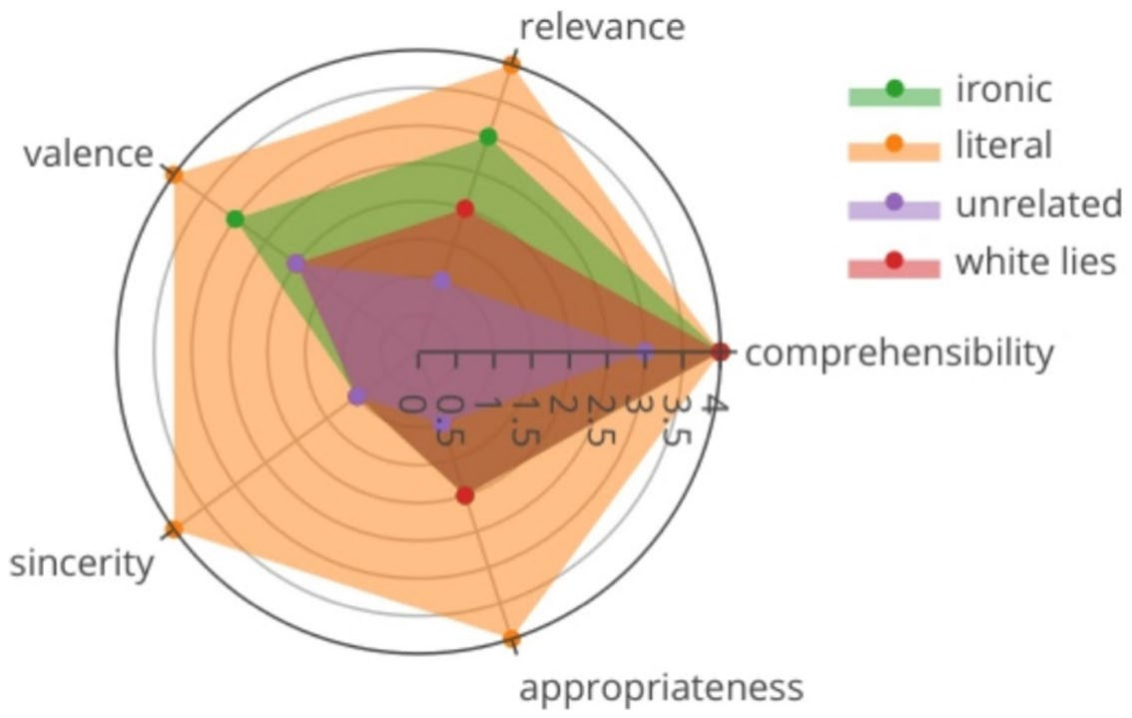
Radar chart showing the psycholinguistic properties associated with each statement category. The scores range from 1, which means less to 4, which means more. A sample of 120 participants rated the stimuli. All categories met their expected psycholinguistic properties. See text for additional details.

Results indicated that all contexts were comprehensible, and that all categories met the desired psycholinguistic properties according to their operational definition. Percentage of classification for each category was as follows: ironic statements (57.14 ± 49.55), white lies (84.76 ± 35.98), unrelated (86.06 ± 34.68) and literal (95.95 ± 19.73). Regarding the psycholinguistic properties, ironic statements were identified as comprehensible (Mdn = 4, IQR = 0), relevant (Mdn = 3, IQR = 2), insincere (Mdn = 1, IQR = 1), inappropriate (Mdn = 2, IQR = 2), and with neutral emotional valence (Mdn = 3, IQR = 2). Literal statements were rated as comprehensible (Mdn = 4, IQR = 0), relevant (Mdn = 4, IQR = 1), sincere (Mdn = 4, IQR = 0), appropriate (Mdn = 4, IQR = 0), and with positive emotional valence (Mdn = 4, IQR = 0). The unrelated statements were identified as comprehensible (Mdn = 3, IQR = 2), irrelevant (Mdn = 1, IQR = 1), insincere (Mdn = 1, IQR = 1), inappropriate (Mdn = 1, IQR = 1), and with neutral emotional valence (Mdn = 2, IQR = 2). The white lies were rated as comprehensible (Mdn = 4, IQR = 0), relevant (Mdn = 2, IQR = 2), insincere (Mdn = 1, IQR = 0), inappropriate (Mdn = 2, IQR = 2), and with neutral emotional valence (Mdn = 2, IQR = 2; see Figure 2).

Results from the first multinomial logistic regression model analysis (with all the statements) showed that the model in the training dataset had a 68.06% classification accuracy, and the validation dataset had a 59.32% classification accuracy. The statement category with the highest classification accuracy was literal (training = 90.18%, validation = 93.44%), followed by unrelated (training = 78.21%, validation = 79.63%), white lies (training = 77.51%, validation = 76.19%); and ironic had the lowest classification accuracy (training = 9.47%, validation = 10.78%). Because the ironic statements had the lowest classification accuracy, in order to try to increase the accuracy, the model was calculated three more times, considering the categories less and more ironic (see 2.1.1.1). The performance of the second model, excluding the more ironic statements, showed that the training dataset had a 73.36% classification accuracy, and the validation dataset had a 63.72% classification accuracy. The performance of the third model, excluding the less ironic statements, showed that the training dataset had a 71.45% classification accuracy, and the validation dataset had a 64.25% classification accuracy. The performance of the fourth model, excluding six ironic statements randomly, showed that the training dataset had a 68.07% classification accuracy, and the validation dataset had a 58.96% classification accuracy. In sum, the model had a 59.32% classification accuracy; accuracy increased when the ironic statements were split into less (63.72%) and more ironic (64.25%), and it decreased when the degree of irony was not controlled (58.96%).

#### Recording of acoustic stimuli to test the effect of prosody

##### Stimulus recording

A total of 40 statements were used including the 14 statements from the contextual discrepancy experiment and 26 new ones created using the previously described methods (see 2.1.1). The statements were recorded by two professional actors, a man and a woman with experience in voice modulation. Each stimulus was recorded by both actors using three different intonations: ironic, literal, and unrelated. For ironic statements, the actors were asked to read with an ironic intonation; for literal statements, they were asked to read as if they really believed what the statements said; and for unrelated statements, the actors were asked to read without intonation. A total of 240 statements were recorded. To select the stimulus that had the expected intonation, two the judges were the coauthors E.V and C.I, and they were blinded to the classification of the statements. The judges classified the intention of the stimuli. Of the 240 audios, 57 were excluded because they did not meet the expected intonation, according to the judges. Of the 183 remaining audios, 47 were judged as ironic (23 female voices), 66 as unrelated (27 female voices), and 70 as literal (37 female voices). This was followed by the evaluation of the acoustic parameters that characterized each intonation.

##### Selection of acoustic parameters

A systematic search was performed to select the relevant acoustic parameters for irony. Following the PRISMA guidelines (Moher et al., 2009) 141 articles that studied the acoustic parameters of irony were identified in the Web of Science (Clarivate *Web of Science*. © Copyright Clarivate 2019) database. The keywords used were “irony” and “sarcasm,” combined with “prosody,” “prosodic,” and “intonation.” Seventy-seven records remained after duplicates were removed. Of the 77 records, 46 did not meet the inclusion criteria, 44 did not associate irony with prosody, and two were chapters of books. Of the remaining 31 articles, nine were excluded because they did not use acoustic markers (7 articles), one was a review, and another did not use prosodic modulation (1 article). Based on the 22 remaining articles we found that in terms of F0, six articles reported a lower F0, six found differences in range, and three articles indicated unspecified variations. For the intensity of voice, 12 articles reported an increase in intensity (Rockwell, 2001; Li et al., 2013; Peters et al., 2016; Deliens et al., 2017). Concerning speech rate, 16 articles reported a slower speech rate and three longer syllables. In conclusion, articles that study ironic statements consistently report changes in the F0, intensity, and speech rate. Thus, these parameters were selected as the acoustic parameters for analysis.

##### Acoustic analysis

Once the acoustic parameters had been selected, noise reduction was performed using the noise reduction parameters recommended by the Audacity program (version 2.2.1) (Audacity Team, 2018). The analyses were performed in R (R Core Team, 2020) using the PraatR library (Albin, 2014), which carries out the analysis from Praat (version 6.0.37) (Boersma and Weenink, 2021). From the 183 audios the median and range were extracted for the F0 (Hz) and intensity (in decibels, dB), also speech rate was calculated by dividing the duration of the audio (seconds, s) by the number of words in the linguistic stimulus.

**Figure 3:**
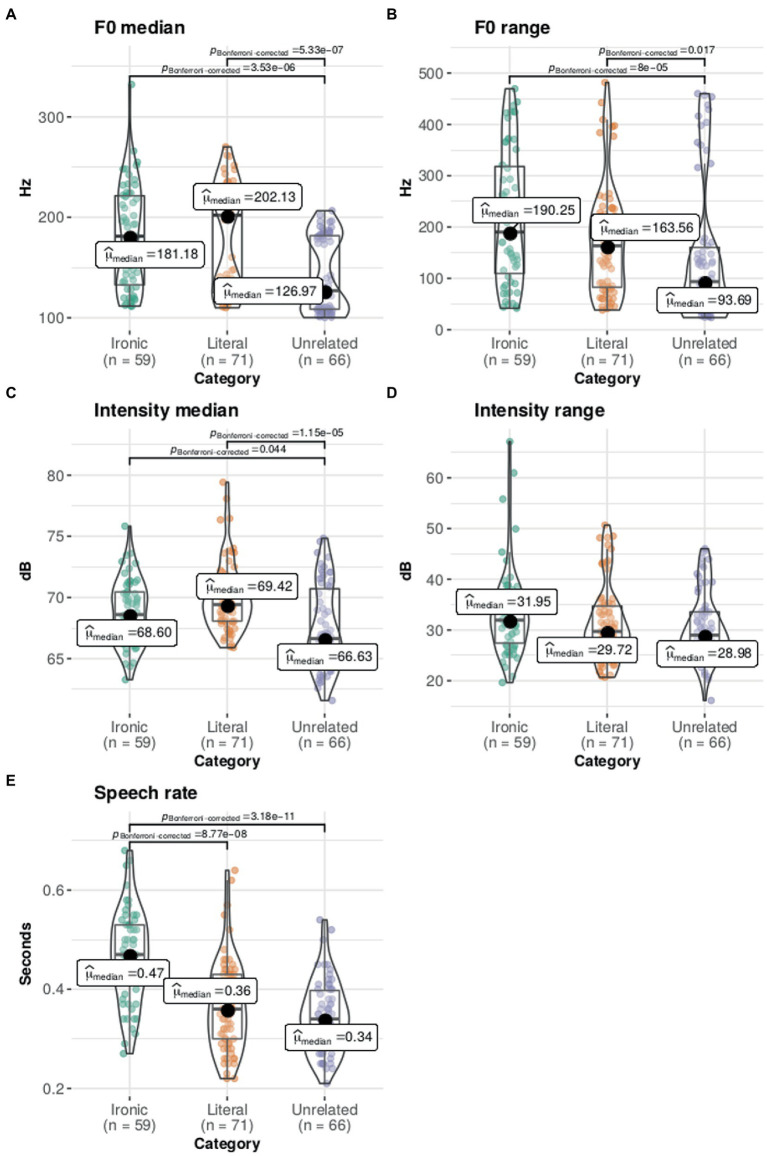
Acoustic parameters by statement category. The statement categories can be differentiated by their acoustic pattern. Panels **(A, B)** show the median and range for the fundamental frequency (F0) in Hz. Panels **(C, D)** show the median and range for intensity in decibels. Panel **(E)** shows the speech rate (duration in s/number of words). Plots show the density curves and the box plots show the median (dark circle), mean (thick line), interquartile range (rectangle), and the lower/upper adjacent values (black lines stretched from the rectangle), and scatter plot. Significant differences between categories are indicated.

A Kruskal–Wallis test followed by Dunn’s test of multiple comparisons with Bonferroni correction showed that there were differences between statement categories in median F0 (H(2) = 54.19, *p* < 0.001), F0 range (H(2) = 15.68, *p* < 0.01), median intensity (H(2) = 16.58, p < 0.01), and median speech rate (H(2) = 51.26, *p* < 0.001). Intensity range did not show significant differences. The pairwise comparisons (see Figure 3) showed significant differences (*p* < 0.01) in F0 medians between ironic and unrelated statements, and between literal and unrelated. For the F0 range, there were differences between ironic and unrelated statements. Likewise, for median intensity, differences were found between ironic and unrelated and between literal and unrelated (*p* < 0.001). For intensity range there were no differences between statements. And for mean speech rate, there were differences between ironic and literal, and between ironic and unrelated (*p* < 0.001). The results indicate that the statements can be indeed distinguished by their acoustic patterns. More specifically, F0, intensity and speech rate distinguish the ironic intonation from the unrelated, while speech rate distinguishes the ironic from the literal intonation (see Figure 3).

#### Selection of facial expressions

##### Materials and procedure

To select the facial expressions that are typically associated with ironic statements, a systematic search was performed. Following the PRISMA guidelines (Moher et al., 2009), 17 records were identified in the Web of Science (Clarivate *Web of Science*. © Copyright Clarivate 2019). The keywords used were “irony” and “sarcasm,” combined with “facial expression.” Thirteen records remained after duplicates were removed. Five of those 13 records studied facial expressions in irony detection. The main search results showed that smiling, arched eyebrows, blank eyes, winking, squinting eyes, and tongue in cheek have been associated with ironic statements (Rockwell, 2001; Attardo et al., 2003; Caucci and Kreuz, 2012).

These gestures were matched by similarity with a facial expression database (Du et al., 2014) that quantified facial expressions using a set of action units. The facial expressions are identified by codes, the code is given by the action units (AU). These AU are movements of individual muscles or groups of muscles associated with the performance of a facial expression. For example, the arched eyebrows are described by the codes by AU 1 and 2, and are used in the expressions happily surprised, disgustedly surprised, among others. Facial expressions that included the AU with greatest similarity to the gestures associated with ironic statements were selected. The AU that matched the facial expressions (e.g., smiling, arched eyebrows) associated with ironic statements were: 1, 2, 4, 5, 12, 24, and 25 (for details see: Du et al., 2014).

The facial expressions that included more AU associated with ironic statements were facial expressions labeled as angrily disgusted, happily surprised, disgustedly surprised, disgusted, and happily disgusted. From the same database, the control facial expressions labeled as blank face, sad and happy were selected. The selection of the actors (two female and two male) was based on the accuracy in which they were recognized by a Mexican sample in a previous study (82.56 ± 6.23) (Rasgado-Toledo et al., 2021). The five experimental and three control facial expressions, from these four actors, were used. The facial expressions were combined with the 14 statements described under Construction of the linguistic stimuli section (see 2.1.1) resulting in 448 combinations (4 actors x 14 statements x 8 facial expressions).

To select the facial expressions associated with ironic statements, the 448 combinations described above (see 2.1.3) were distributed in six Google Forms surveys. The facial expression was presented at the top of the page, while the statement was presented below. On the lower part of each page participants were asked to classify the intention of the statement according to the facial expression. The options were: ironic, literal, unrelated, white lies, and none.

##### Participants

Participants were asked to fill in a general data form with information about their level of education, sex, and age. The six surveys were answered by 132 participants (77F, 55 M, 1 n.d.), with a mean age of 26.22 ± 4.9. All of them were native Spanish-speakers and undergraduate or graduate students that did not report any psychiatric or neurological disorders.

##### Results

**Figure 4:**
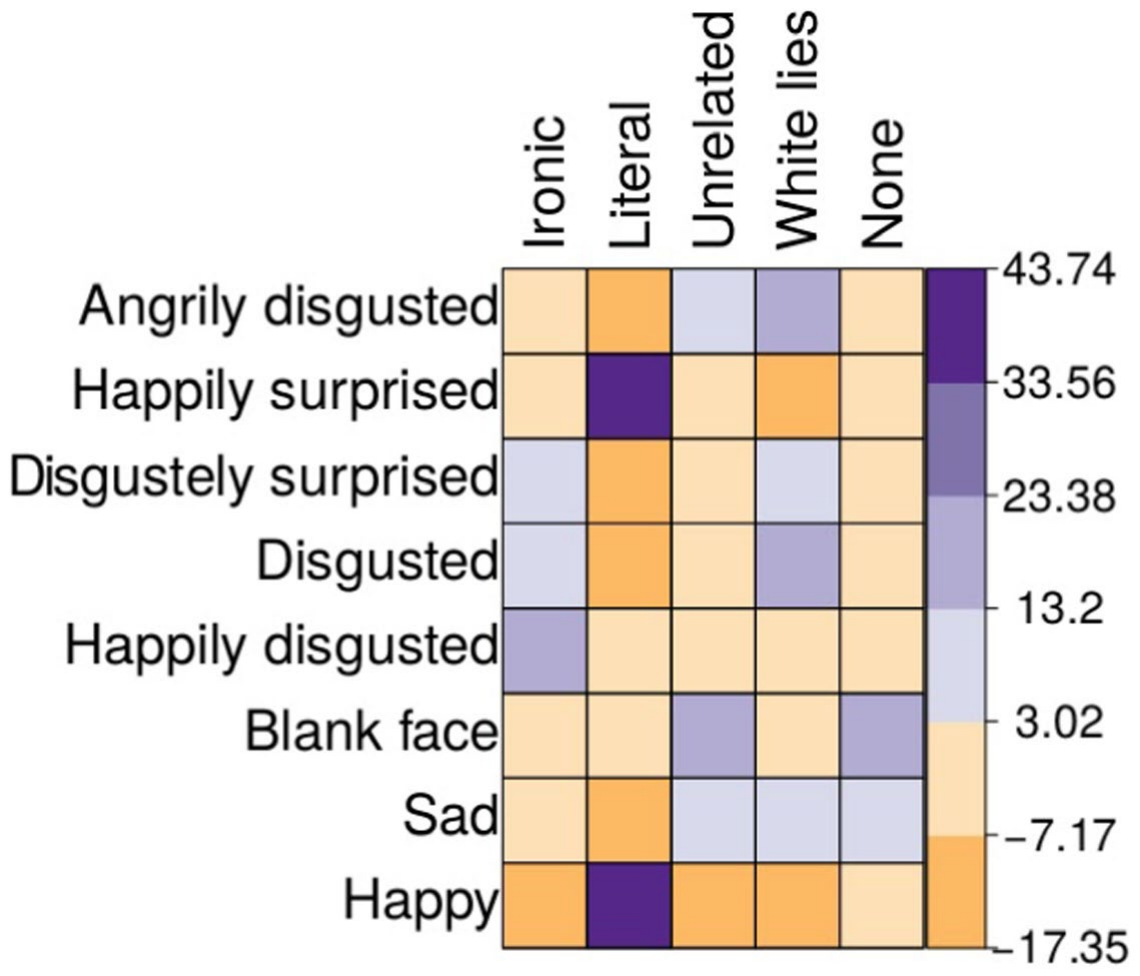
Mosaic plot showing the strength of the association between categories of statements and facial expressions based on the results of Pearson standardized residuals obtained from a chi-squared test. Those facial expressions that had a greater association (deeper hue) with each category were selected: happily disgusted for ironic statement, happy for literal, blank face for unrelated, and disgusted for white lies. Warm colors indicate a negative sign, and cold colors a positive sign for the residuals.

A chi-square test indicated that there was a relationship between the statement’s intention and the facial expressions (*X*^2^ (28,135) = 7401.46, *p* < 0.001). A *post hoc* test with Bonferroni correction showed that the highest associations between intention and facial expressions were between ironic statements and happily disgusted (*p* < 0.001), literal statements and happy (*p* < 0.001), unrelated statements and blank face (*p* < 0.001), white lies and disgusted (*p* < 0.001), and none with blank face (*p* < 0.001; see Figure 4).

### Irony identification

Once all stimuli were selected, a total of 3 different experiments were conducted. Each of the experiments was performed by a different cohort of participants. Participants were asked to sign an informed consent to participate in the study and fill in a general data form with information about their level of education, sex, and age. All participants were native Spanish-speakers, undergraduate or graduate students, and reported no psychiatric or neurological disorders. The project was reviewed and approved by the Ethics Committee (Comité de Ética en la Investigación) of the Insituto de Neurobiología, which follows national and international guidelines (#047.H.RM).

The classification accuracy mean and standard deviation were obtained for the three experiments and represents the percentage of participants that classified each item accurately. For the first experiment, the median and IQR for the classification time, context reading time, and statement reading time were computed. For the second and third experiments, the median and IQR were computed for the classification time. The assumption of normality was assessed with the Shapiro test. Results showed that none of the three experiments met the assumption of normality (*p* < 0.05). The Levine test was performed to evaluate if the variances were equal between categories; the homoscedasticity assumption was not met. Thus, a Friedman test, followed by a Durbin-Conover *post hoc* test with Bonferroni correction, were performed. For all the experiments a Spearman correlation, with FDR correction, was computed for behavioral data and scores from psychometric tests. Statistical significance was set at *p* < 0.05.

#### Experiment 1: Contextual discrepancy as a cue for irony identification

##### Participants

The task was completed by 30 participants (15 females), with a mean age of 22.73 ± 3.63; native Spanish-speakers. All of them were undergraduate or graduate students that did not report any psychiatric or neurological disorders.

##### Materials and procedure

**Figure 5:**
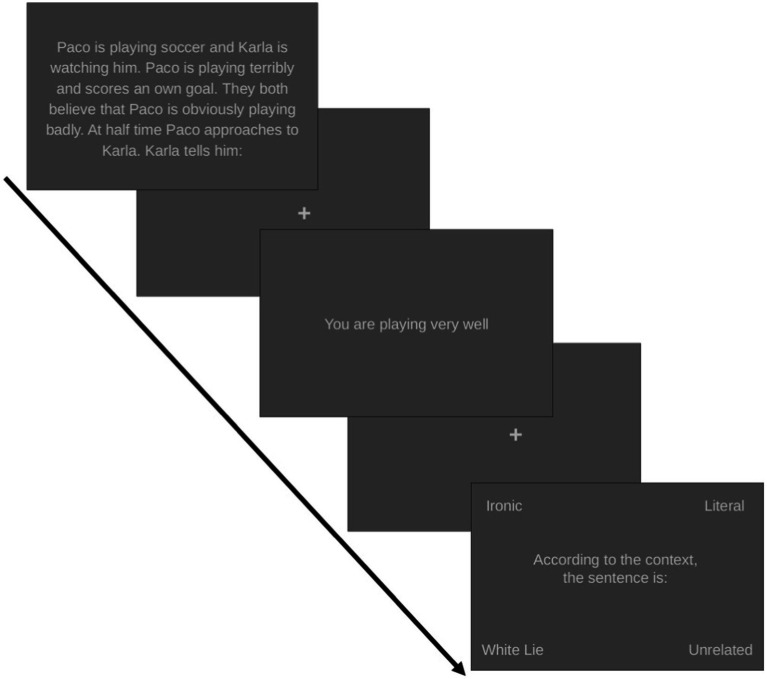
Contextual discrepancy task for Experiment 1, Contextual Discrepancy. The stimuli were presented in text modality.

Once the stimuli were constructed and validated, the next step was to assess if they were correctly identified. For this purpose, a classification task was created using the 56 stimuli created previously (14 by each category) in Psychopy (version 1.82) (Peirce, 2007) (see Figure 1). The first screen contained the social context; the second, the statement; and the third, the following question: “According to the context, the statement is:..,” and four options located in each corner of the screen (see Figure 5). The task was presented using written text. Participants were asked to press the enter button once they finished reading the first and second screens. The third screen changed when they selected their answers. The variables obtained from this task were answers, classification time, also reading time of contexts and statements.

Before starting the task, participants were given the instruction: “You are going to read social contexts where two persons interact. One of them will utter a statement at the end. When you read the statement it is important to try to detect the intention. Then you will be asked to select the intention of the statement according to the context. The four options are: ironic, a statement that is used to joke about something very obvious in the context; literal, a statement that conveys what the speaker really thinks; white lies, statements used to hide the truth; and unrelated, a statement that has no relation to the context. You must press the spacebar to continue. The selection screen will change once you choose an option.``.

Then, participants were requested to complete a psychometric battery that evaluated different cognitive processes. For general intelligence, Raven’s progressive matrices were applied (Raven, 2007). For verbal fluency, the verbal fluency task from the Batería Neuropsicológica de Funciones Ejecutivas (BANFE) (Flores et al., 2011) was used. ToM was evaluated with the Short Story Task (SST) (Dodell-Feder et al., 2013; Giordano et al., 2019). Perceptual reasoning was evaluated with the block design test, and working memory with the Digit Span Forward and Backward subtest of the Wechsler Adult Intelligence Scale (WAIS) (Wechsler, 2007).

##### Results

**Figure 6:**
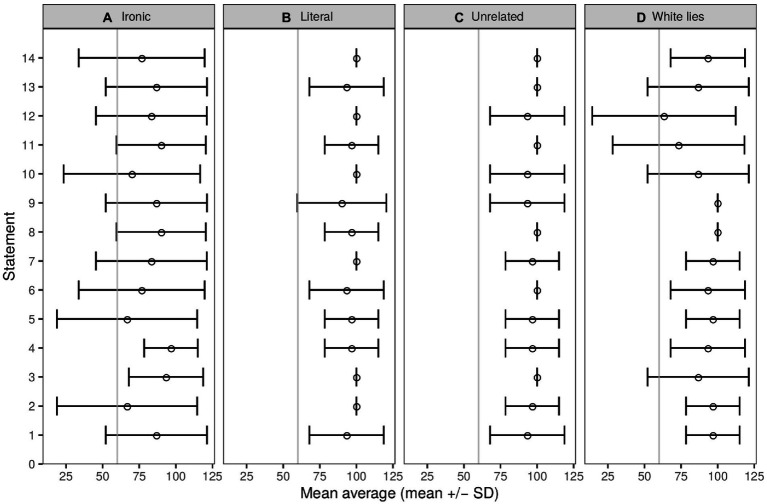
Classification accuracy for each statement for Experiment 1 that explored the effect of contextual discrepancy on the identification of irony. Classification accuracy represents the percentage of participants that classified each item accurately (mean + sd), according to its category **(A)** Ironic, **(B)** Literal, **(C)** Unrelated, **(D)** White lies. The vertical gray line indicates the 65% classification accuracy, which was the minimum for inclusion. All statements met the inclusion criteria.

The percentage of classification was as follows: 82.38 ± 38.14 for ironic statements, 90.24 ± 29.72 for white lies, 96.91 ± 29.72 for literal statements and 97.14 ± 18.68 for unrelated statements. The faster median classification time (seconds) for correctly identified statements was 1.80 (IQR = 0.88) for literal, 1.97 (IQR = 1.20) for white lies, 1.99 (IQR = 1.17) for unrelated, and 2.27 (IQR = 1.99) for ironic. The faster median context reading time (seconds) was 10.93 (IQR = 6.96) for literal statements, 11.34 (IQR = 8.60) for unrelated statements, 11.81 (IQR = 7.88) for white lies, and 12.35 (IQR = 7.58) for ironic statements. The faster median statement reading time (seconds) was 1.25 (IQR = 0.88) for literal, 1.40 (IQR = 1.03) for white lies statements, 1.40 (IQR = 1.26) for ironic, and 1.52 (IQR = 0.93) for unrelated (for details see: Supplementary Table 1). Each item was accurately classified by at least 65% of participants (see Figure 6).

Results indicated that there were significant differences among statement categories in terms of classification accuracy (*X*^2^_Friedman_(3) = 28.10, *p* < 0.001), classification time (*X*^2^_Friedman_(3) = 25.08, *p* < 0.001), context reading time (*X*^2^_Friedman_(3) = 9.36, *p* < 0.05), and statement reading time (*X*^2^_Friedman_(3) = 11.80, *p* < 0.01). The *post hoc* tests showed significant (*p* < 0.05) differences in classification accuracy between ironic with literal, unrelated, and white lies (*p* < 0.05). Differences in classification time were between ironic with literal, unrelated, and white lies. For context reading time there was a significant difference between irony and literal. For statements reading time there were significant differences between literal with irony (*p* < 0.05), and literal with unrelated (*p* < 0.05).

Concerning the classification task and psychometric tests, irony classification time had a negative correlation with the comprehension scale from the SST (rho = −0.45, *p* = 0.05). Irony context reading time had a positive correlation with the spontaneous mental state inference scale from the SST (rho = 0.45, *p* = 0.05). The white lies context reading time had a negative correlation with digit span forward (rho = −0.48, *p* = 0.05) and digit span backward (rho = −0.52, *p* = 0.05). The white lies statements reading time had a positive correlation with the spontaneous mental state inference scale (rho = 0.56, *p* = 0.01). The unrelated context reading time had a positive correlation with spontaneous mental state inference scale (rho = 0.51, *p* = 0.05) and negative correlation with digit span forward (rho = −0.49, *p* = 0.05). The unrelated statements’ reading time had a positive correlation with spontaneous mental state inference scale (rho = 0.47, *p* = 0.05; see Supplementary Figure 1).

##### Stimulus recording

We found that the stimuli were correctly identified but that reading times for contexts and statements presented great variability. This was a significant finding since the purpose of this paper was to design a task for neuroimaging studies. Thus, we decided to audio-record the stimuli to reduce this variability in reading speed among the participants. The recording was made in wav format, in a noise-free room, and without distracting stimuli. Then, the social contexts and statements were recorded by a female and a male voice, without modifications in F0, intensity, or speed. A total of 140 audios were recorded.

##### Acoustic analysis

Noise reduction was done using the noise reduction parameters recommended by the Audacity program (version 2.2.1) (Audacity Team, 2018). The analysis was performed in R (R Core Team, 2020), using PraatR library (Albin, 2014), which carries out the analysis from Praat (version 6.0.37) (Boersma and Weenink, 2021). Because the statements were the same in the four categories, only contexts were compared. The mean and range of the F0, intensity, and audio duration parameters were extracted for each context and statement. Speech rate was obtained by dividing the audio duration by the number of words in the linguistic stimulus.

To compare the acoustic parameters among context categories, a Kruskal-Wallis test followed by pairwise comparisons using Wilcoxon tests with Bonferroni correction, were performed. There were no differences in the acoustic parameters among context categories in F0 median, F0 range, intensity median, range intensity, or speech rate. The *post hoc* tests corroborated that there were no differences between contexts.

#### Experiment 2: Prosody and facial expression as cues for irony identification

##### Participants

The tasks were completed by 30 adults (15 female), with a mean age of 28 years (21–40 years); native Spanish-speakers. All of them were undergraduate or graduate students that did not report any psychiatric or neurological disorders.

##### Materials and procedures

To evaluate the identification of ironic statements using the cues provided by prosody and facial expression, a task for each type of cue was created. Because white lies were not associated with a specific facial expression (see Figure 4), they were excluded from the following experiments. The ironic, literal, and unrelated categories were used for both cues. The prosody and facial expression tasks were created in Psychopy Pavlovia (version 3.0.2) (Peirce et al., 2019). Stimuli were randomly presented. Applications were made online through Psychopy Pavlovia (3.0.2 version) (Peirce et al., 2019). The cues were evaluated separately by the same sample of participants.

Additionally, participants completed a battery of tests including the Reading the Mind in the Eyes test, that measures ToM (RMET) (Baron-Cohen et al., 2001a); the Autism Spectrum Quotient, that measures abilities associated with autism (i.e., social skills, communication, attention to detail, attention switching, imagination) and has demonstrated to be sensitive in neurotypical population (AQ) (Baron-Cohen et al., 2001b); and the Sarcasm Self-Report Scale that measures how frequent sarcasm is used (SSS) (Ivanko et al., 2004). The AQ and the SSS were applied using Google Forms.

###### Prosody

**Figure 7:**
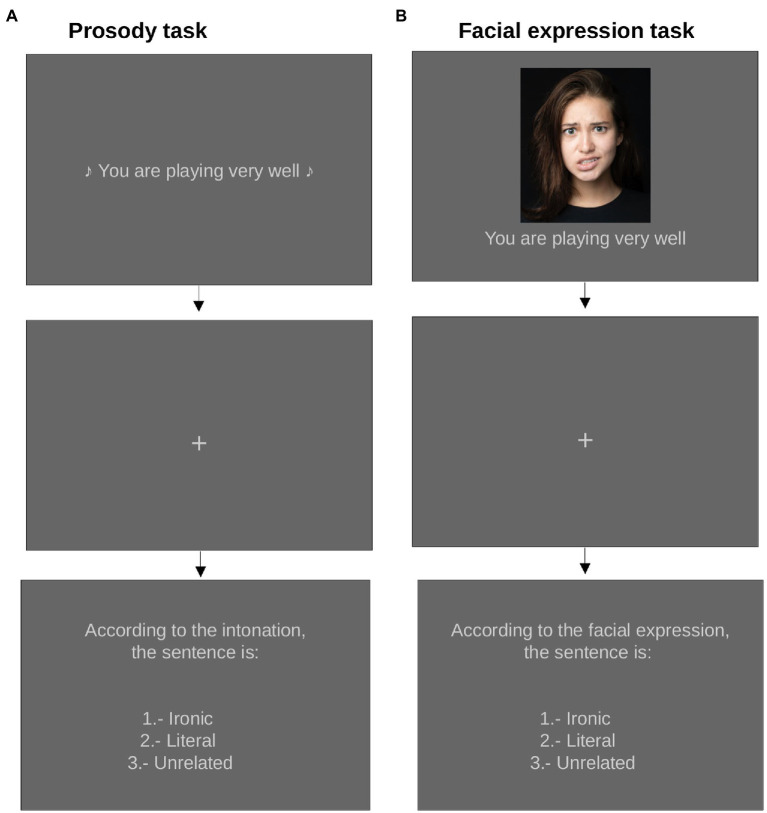
Prosody and facial expression tasks (Panel **A,B**, respectively). For both tasks stimuli were split into two versions, counterbalancing female and male voices or faces. The first slide presented the statement, then a fixation cross was shown for 1 s, followed by a slide that asked the participant to classify the statement heard or read. The photograph presented in panel **(A)** is an illustrative image (photo by Ospan Ali, available from Unsplash; https://unsplash.com). For the experiment, we used the images from Du et al. (2014) with permission from the authors.

For the prosody task, 183 audios that met the required acoustic characteristics according to their statements categories, were selected. Participants heard the statements with different intonations, then were asked to classify the intent of the statement according to its prosody (i.e., ironic, literal, or unrelated). On the first screen, the statement was presented in audio modality and had a fixed duration of 2 s. A fixation cross, with a duration of 1 s, separated the first and second screens. On the second screen, participants were asked to classify the statement according to its intonation (prosody); the options were presented as a list (1.- ironic, 2.- literal, 3.- unrelated). To continue, they had to select one of the three options (see Figure 7A). The various statement categories were randomly presented.

Before starting the task, participants were given the instruction: “You are going to hear statements with different intonations. When you hear the statements it is important to try to detect the intention. You will be asked to select the intention of the statement according to the intonation. The options are: ironic, a statement used to joke about something very obvious; literal, a statement that conveys what the speaker really thinks; and unrelated, a statement that has no intention. The selection screen will change once you choose an option.”

###### Facial expression

For the facial expression task, the three facial expressions that had previously shown the greatest association with the statements categories of interest were used; i.e., happily disgusted for ironic statements, happy for literal, and blank face for unrelated. The 40 statements that were previously designed (120 stimuli = 3 facial expression x 40 statements) were used for this experiment. The facial expression together with the written statement were presented on the first screen. Participants had to press the spacebar to continue to the next screen. After a one-second fixation cross was presented, the second screen appeared and the participants were asked to classify the statement according to the accompanying facial expression. The options were presented as a list (1.- ironic, 2.- literal, and 3.- unrelated). To continue, they had to select one of the three options (see Figure 7B). The various statement categories were randomly presented.

Before starting the task, participants were given the instruction: “You are going to see faces with different facial expressions accompanied by statements. When you see the facial expression and statement it is important to try to detect the intention of the statement according to the facial expression. You will be asked to select the intention. The options are: ironic, a statement used to joke about something very obvious; literal, a statement that conveys what the speaker really thinks; and unrelated, a statement that has no intention. The selection screen will change once you choose an option.”

##### Results

**Figure 8:**
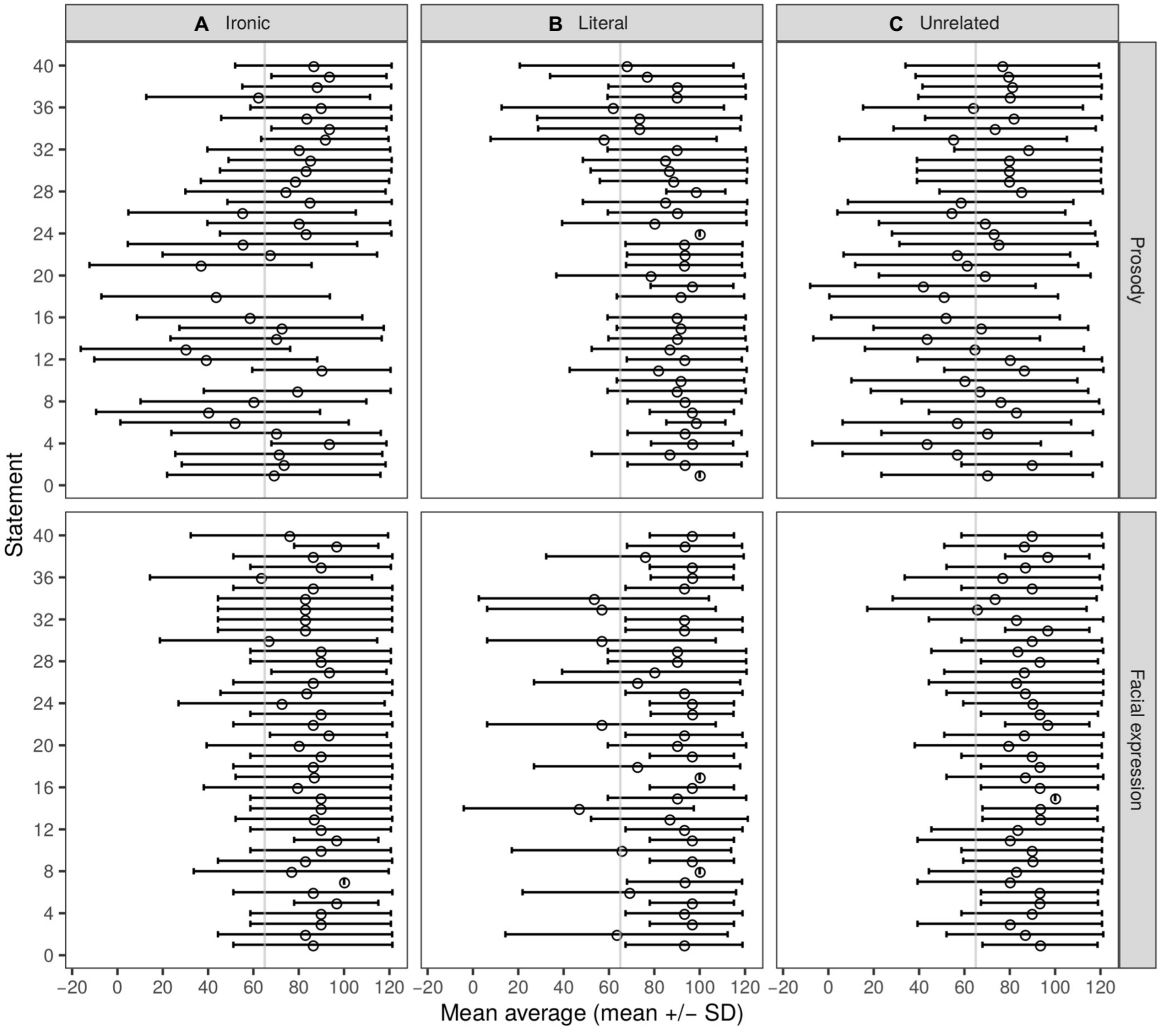
Classification accuracy for each statement for Experiment 2 that explored the effect of Prosody (upper panels) and Facial expression (lower panels) on the identification of irony. Classification accuracy represents the percentage of participants that classified each item accurately (mean + sd), according to its category **(A)** Ironic, **(B)** Literal, **(C)** Unrelated. The vertical gray line indicates the 65% classification accuracy, which was the minimum for inclusion.

For subsequent analyses, only stimuli that were accurately classified by 65% or more of participants were used (see Figure 8). The following stimuli met the criteria for the prosody cue: 42 of the 59 ironic stimuli (22 female voice); 69 of the 71 literal stimuli (36 female voice); and 42 of the 66 unrelated stimuli (17 female voice). For the facial expression cue, the following met the criteria: 39 of the 40 ironic stimuli (19 female faces), 34 of the 40 literal stimuli (15 female faces), and all the unrelated stimuli met the criteria.

For the prosody task, the classification accuracy was 80.40 ± 18.06 for ironic, 88.43 ± 10.29 for literal, and 78.50 ± 22.17 for unrelated statements. For the facial expression task, the classification accuracy was 85.07 ± 18.13 for ironic, 90.57 ± 11.43 for literal, and 87.33 ± 15.15 for unrelated statements. In the prosody task, the median classification time (seconds) by category of statement was 0.45 (IQR = 0.32) for ironic, 0.44 (IQR = 0.72) for literal, and 0.41 (IQR = 0.54) for unrelated statements. In the facial expression task, the classification time (seconds) was 0.38 (IQR = 0.55) for ironic, 0.38 (IQR = 0.48) for literal, and 0.41 (IQR = 0.61) for unrelated statements (for details see: Supplementary Table 2).

The correlation analysis showed that the classification time for literal statements in the prosody task, had a positive correlation with classification time in the RMET (rho = 0.57, *p* = 0.01). The classification time for unrelated statements in the facial expression task, had a negative correlation with the imagination subscale of the AQ (rho = −0.49, *p* = 0.05, see Supplementary Figure 2).

#### Experiment 3: Comparison among contextual discrepancy, prosody, and facial expression on classification accuracy and time of irony identification

The results of the two previous experiments showed that contextual discrepancy, prosody, and facial expression allowed participants to correctly identify the statements’ categories, i.e., irony, literal, unrelated, and white lies. The next step was to compare classification accuracy and latency between cues to evaluate which cue best conveyed the intentions of interest. Therefore, we designed a third experiment using the three cues and the three statement categories in the same sample of participants.

##### Participants

The task was completed by 30 native Spanish-speakers (17 female). The mean age was 27.26 ± 5.06. All of them were undergraduate or graduate students that did not report any psychiatric or neurological disorders.

##### Materials and procedure

The contextual discrepancy task was similar to the one described in Experiment 1, with two differences. The first was that the stimuli were presented in audio modality. The second, was that white lies were not included to homogenize the statement categories between the three tasks. The tasks for prosody and facial expression cues were similar to those described previously, the only difference was that only stimuli that had 65% or higher classification accuracy were used. Stimuli were randomly presented. The application was completed in Psychopy through Zoom (v5.7.7; Zoom Video Communications, Inc., 2021; see Figure 1), which allowed us to give participants remote access to the task and execute Psychopy on the local computer. The participants instructions were the same as previously described.

Additionally, participants performed the SST (Dodell-Feder et al., 2013) and RMET (Baron-Cohen et al., 2001a) to evaluate ToM during the online session. Prior to the online session, the SSS (Ivanko et al., 2004) and AQ (Baron-Cohen et al., 2001b) were answered through a survey created using Google forms.

##### Results

**Figure 9:**
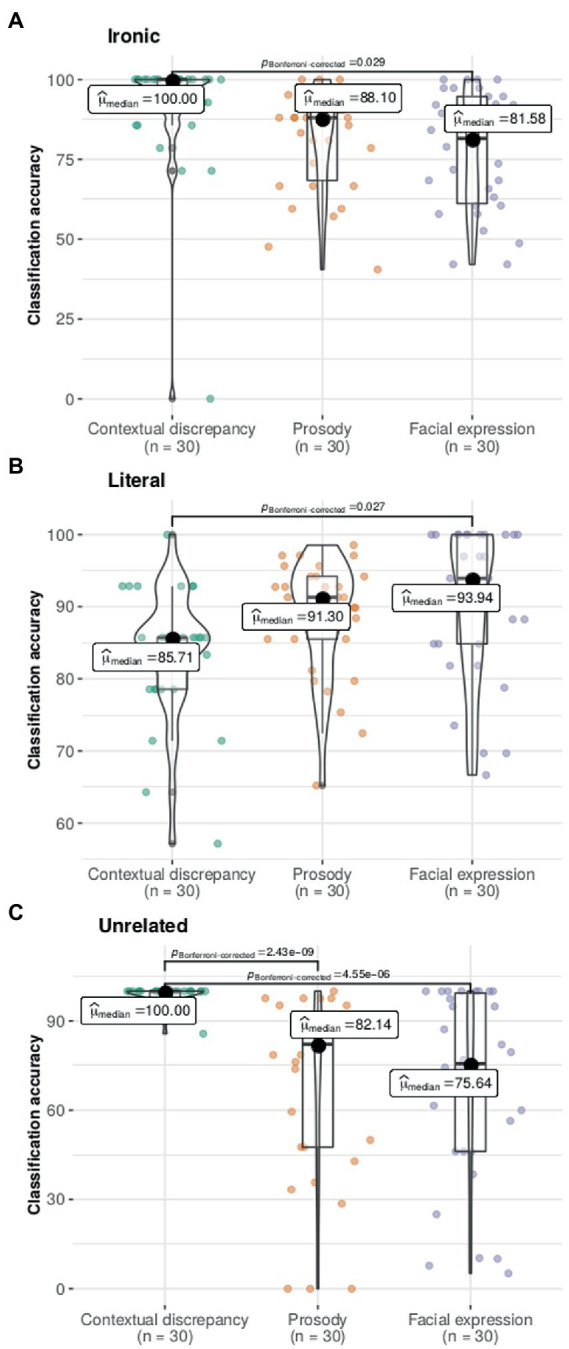
Experiment 3. Classification accuracy for each statement category depending on the type of cue. Classification accuracy represents the percentage of participants that classified each item accurately. The contextual discrepancy resulted in significantly greater classification accuracy for ironic (Panel **(A)**) and unrelated statements (Panel **(C)**) whereas for literal statements (Panel B) facial expression led to greater accuracy. Plots show the density curves and the box plots show the median (dark circle), mean (thick line), interquartile range (rectangle), and the lower/upper adjacent values (black lines stretched from the rectangle), and scatter plot. Significant differences between cues are indicated.

There were significant differences in classification accuracy, depending on the type of cue, for ironic (*X*^2^_Friedman_(2) = 7.13, *p* < 0.05), literal (*X*^2^_Friedman_(2) = 6.87, *p* < 0.05) and unrelated (*X*^2^_Friedman_(2) = 29.89, *p* < 0.001) statements. The pairwise comparisons showed that for ironic statements contextual discrepancy resulted in greater accuracy than facial expression, and similar accuracy to prosody. In contrast, for literal statements contextual discrepancy resulted in significantly lower accuracy compared to facial expression, and similar accuracy to prosody. For unrelated statements contextual discrepancy led to greater accuracy than the other two cues (Figure 9).

The median of classification time (seconds) for ironic statements was 2.89 (IQR = 1.50) for the contextual discrepancy, 2.06 (IQR = 1.08) for prosody and 1.62 (IQR = 0.77) for facial expression. For the literal statement the classification time was 2.50 (IQR = 1.27) for the contextual discrepancy, 1.96 (IQR = 0.97) for prosody and 1.50 (IQR = 0.46) for facial expression. Furthermore, for the unrelated statements classification time was 2.56 (IQR = 1.27) for the contextual discrepancy, 1.92 (IQR = 0.71) for prosody and 1.70 (IQR = 0.99) for facial expression (for details see: Supplementary Table 3).

For classification time, there were significant differences between cues for ironic (*X*^2^_Friedman_(2) = 31.27, *p* < 0.001), literal (*X*^2^_Friedman_(2) =32.47, *p* < 0.001) and unrelated statements (*X*^2^_Friedman_(2) =8.47, *p* < 0.05). The pairwise comparisons showed that, for all statement types, classification times were higher for the contextual discrepancy compared to the other cues. In addition, for literal statements classification times were slower for prosody than facial expression.

The correlation analysis showed that the accuracy of irony identification with the prosody cue had a negative correlation with the scale of social skills from the AQ (rho = −0.51, *p* = 0.05). For the facial expression cue, the classification accuracy of unrelated statements had a positive correlation with SSS (rho = 0.54, *p* = 0.05; see Supplementary Figure 3).

## Discussion

This study aimed to design a task to evaluate irony comprehension in Spanish speakers and to correlate irony comprehension with cognitive functions. The results show that we were able to design a task that may be used in neuroimaging studies to test irony comprehension using different cues, contextual discrepancy, prosody, or facial expressions. The contextual discrepancy was tested using text and audio recordings to reduce the variability in reading times. We found that the contexts and statements selected were comprehensible and had the expected psycholinguistic properties according to the type of statement, i.e., literal, ironic, unrelated, and white lies. When comparing the three types of cues, we found that the best cue for irony comprehension was the contextual discrepancy. However, both ironic prosody and facial expression resulted in correct identification and required lower classification time. All experiments were tested with adult participants, but the scenarios are compatible with situations that adolescents might experience and thus may be used with this age group. With regard to children, although by 6 years of age children can understand irony (Glenwright and Pexman, 2010), the tasks should be piloted first because the situations may depicted may not be easy to understand.

The task that we present allows for the systematic evaluation of each cue’s role separately compared to other tasks in neuroimaging (fMRI) studies (see Reyes-Aguilar et al., 2018). In general, have studies used contextual discrepancy as a cue, i.e., written material followed by an ironic or non-ironic utterance and occasionally prosody, no study used facial expression as a cue. Thus, this task can evaluate irony comprehension in Spanish speakers using the cue of interest (the different versions of the task are available in Pavlovia, please see the data availability section).

According to Attardo (2000), the psycholinguistic properties of ironic statements are relevance, are inappropriateness to the context, and are used by the speaker to convey the true meaning to the listener. In our first experiment using contextual discrepancy as the cue of interest, we found that a multinomial logistic regression analysis could classify the type of statement (i.e., ironic, literal, unrelated, white lies) possible based on the ratings of the psycholinguistic properties of relevance, appropriateness, and sincerity provided by the participants. The accuracy of classification increased when the degree of irony was considered. We also found that ironic statements had the lowest classification accuracy and the longest classification time. Also, despite no differences in context or statement length, ironic statements required longer reading time, for both context and statements, compared to literal statements. Together, these results could reflect a significant difficulty in detecting ironic statements, which agrees with the proposal that ironic statements are one of the most complex pragmatic forms to interpret (Wilson and Sperber, 1981).

In terms of the acoustic characteristics of ironic prosody, we found that classified as ironic stimuli had a slower speech rate than literal and unrelated statements, in agreement with previous studies (Rockwell, 2001; Cheang and Pell, 2009; Bryant, 2010; Li et al., 2013; Peters et al., 2016; Voyer and Vu, 2016; Deliens et al., 2018). Ironic stimuli had higher median intensity and higher fundamental frequency (F0) (median and range) than unrelated statements only; others have found that ironic stimuli have greater intensity (Rockwell, 2001; Li et al., 2013; Peters et al., 2016; Deliens et al., 2018). Although the prosody of ironic statements had a lower fundamental frequency (F0) than literal statements, this difference was not statistically significant as expected based on the literature (Rockwell, 2001; Li et al., 2013; Peters et al., 2016; Deliens et al., 2018). One possibility is that the specific acoustic conventions to express irony may differ between languages, as has been suggested (Rockwell, 2001; Cheang and Pell, 2009; Bryant, 2010; Li et al., 2013; Peters et al., 2016; Voyer and Vu, 2016; Deliens et al., 2018). Another is that the acoustic correlates of ironic prosody are not intrinsic but relative to the enfolding discourse (Bryant, 2010). Still, as was expected, we found that acoustic parameters can distinguish between statements categories; the ironic stimuli can be distinguished by speech rate from the literal and unrelated statements and by intensity and F0 from unrelated statements. Only statements correctly classified by at least 65% of the participants were selected for the last experiment.

Regarding facial expression, we found that happily disgusted was the facial expression most closely associated with ironic statements. This facial expression has AUs that match the variations previously associated with irony, such as smiling, arched eyebrows, and squinting eyes (Rockwell, 2001; Attardo et al., 2003; Caucci and Kreuz, 2012). Additionally, the literal statements were associated with a happy face and the unrelated statement with a blank face. These results indicate that the three statement categories can be reliably associated with specific facial expressions and are identified as intended. Only stimuli that were correctly classified by at least 65% of the participants were selected for the last experiment.

Since we aimed to create a reliable task of irony comprehension for neuroimaging studies, in the last experiment, we compared accuracy and classification time between cues. The results showed that ironic and unrelated statements were more accurately detected when the contextual discrepancy was present, in agreement with Deliens et al. (2018) findings. The literal statements were more accurately detected when the facial expression was available, although they were also accurately detected with contextual discrepancy or prosody. These results show that despite the three cues successfully transmitting the intended meaning, contextual discrepancy seems to be a better cue for transmitting the ironic message. Another advantage of contextual discrepancy as a cue is that it allows more flexibility in modifying the message and the comparison to other categories of statements, such as white lies, as was done in the first experiment.

We found that classification time for all categories of statements was higher for contextual discrepancy compared to the other cues. Deliens et al. (2018) found similar results in a task that used videos as stimuli; they suggested that cognitive economy principles drive reliance on ironic prosody or facial expression at the expense of a more reliable but costlier option, contextual processing. EEG experiments have reported that recognition of meaning occurs early when prosody or an emoji are used as cues, this is reflected in an increase in the P200 potential (Regel et al., 2010, 2011; Wickens and Perry, 2015; Weissman and Tanner, 2018). Also, studies that used prosody without context have reported the absence of the P600 potential, which is associated with integration and reanalysis (Cornejol et al., 2007; Wickens and Perry, 2015; Gibson et al., 2016). These results support the cognitive economy principle suggested by Deliens et al. (2017), and may explain the shorter classification times in the presence of prosody and facial expression cues we found.

Our secondary aim was to correlate irony comprehension with Theory of Mind, frequency of use of sarcasm, and the Autism Spectrum Quotient (this scale measures abilities associated with autism, such as social skills and imagination, but has been demonstrated to be sensitive in neurotypical population, by Baron-Cohen et al., 2011). However, we found an inconsistent association between irony comprehension and these tests. In the first experiment-contextual discrepancy-we found a negative correlation between irony classification time and the comprehension scale from the short story task. This scale evaluates language comprehension in general. Interestingly, in this experiment in which context and statements had to be read, we found significant positive correlations between context and statement reading times for ironic, unrelated and white lies categories and the spontaneous mental inference for the short story task. These results suggest an association between the cognitive effort exerted to understand non-literal written material and the ability to understand the mental state of others. In this experiment we also included general domain tests. However, we found only an association between reading times and working memory for unrelated statements and white lies, and thus we did not include these measures in the subsequent experiments.

In the second experiment-prosody and facial expression-we did not find any significant correlations between accuracy or classification time for ironic stimuli and tests of social cognition; the only correlations we found were with classification time for literal and unrelated statements. In the last experiment comparing the three cues, we found a significant negative correlation between the accuracy of irony identification when using the prosody cue and the scale of social skills from the Autism Quotient test; and a positive correlation between classification accuracy of unrelated statements when using the facial expression cue and the scale that measures the frequency of sarcasm use.

These inconsistent results reflect the difficulty in finding cognitive correlates of irony comprehension. The reason may be that a variety of skills are necessary, from language skills to social cognition, including the theory of mind, identification of emotions, and social experience (Pexman et al., 2019; Fabry, 2021). Another possibility is that the lack of association could be due to relatively uniform scores between participants in those experiments. A broader and more heterogeneous sample of participants may be needed to fully assess this association (Baron-Cohen et al., 2001b).

While our primary purpose was to develop a reliable task of irony comprehension for Spanish speakers, our results may also contribute to assessing the theoretical models. If we consider classification time overall, we find that classification of irony takes longer when using contextual discrepancy as a cue but that this leads to greater accuracy in irony detection. These results support the standard pragmatic view, which proposes that once a listener detects an ironic statement, she first constructs the literal interpretation. When it becomes apparent that the literal interpretation is incompatible with the context, the listener computes the ironic interpretation, which requires more time for the receptor (Grice, 1975). Also, according to the standard pragmatic view, ironic interpretation requires more effort and resources (Grice, 1975). However, when using prosody or facial expression as cues, we find that classification time of ironic stimuli drops, but so does accuracy. These results support the direct access view (Gibbs, 1994) and the graded salience hypothesis (Giora, 1997), in that an ironic interpretation is activated directly, either because the context or the salient cues support it.

When all cues are presented simultaneously, as in the Deliens et al. (2018) study, prosody and facial expression were associated with shorter response times, regardless of the presence or absence of a context. These cues did not have a cumulative effect on the context because there was no greater accuracy when all three cues were present. The authors propose that salient cues, i.e., prosody and facial expression, are privileged by interpreters whenever possible. The failure to see that a context-based assessment is more reliable than other cues may be considered a meta-cognitive error. These results and those of the present experiment appear to support Pexman’s constraint satisfaction model (2008), which proposes that cues activated by a statement are processed rapidly and in parallel. Once there is sufficient evidence, an ironic interpretation is given. Which cues are privileged would likely depend on the interplay between the scenario and the experience with irony by the interlocutors.

The limitations of the present study are that we evaluated each type of cue separately. Thus, this design does not reflect natural social interactions when all cues are present simultaneously. More dynamic media, such as videos, could help understand how the different cues interact and impact irony identification. For neuroimaging studies, however, videos must be carefully considered because they require the control of multiple variables between conditions. Another issue to consider is the number of statement categories to include. We chose to include white lies in the first experiment because the literature suggests that this category is often confused with irony (e.g., Pexman, 2008); however, it does not have associated prosody or facial expression. Therefore, we could not include this category when we compared the various cues. In the last experiment, the change in the number of options appeared to improve irony comprehension accuracy. Thus, this is a variable that should be considered in future studies. Finally, the psychometric battery of tests did not evaluate all the cognitive processes associated with social communication. It may be necessary to include tests that evaluate processing style and executive functions, such as inhibitory control, as well as measures of linguistic abilities, since pragmatic ability may be more related to linguistic competence than to other cognitive variables. Finding out which cognitive and linguistic abilities correlate with pragmatic comprehension is the first step in designing successful interventions for individuals with social communication problems that affect their personal and professional lives.

## Data availability statement

The datasets presented in this study can be found in online repositories. The names of the repository/repositories and accession number(s) can be found at: The code for this study can be found in Github: https://github.com/Eli1404/verbal.irony.behavioral.git. The tasks for the three cues can be found in Pavlovia: https://gitlab.pavlovia.org/Elizabeth14/contextual-discrepancy.git; https://gitlab.pavlovia.org/Elizabeth14/entonacion_i.git; https://gitlab.pavlovia.org/Elizabeth14/expresion-facial_i.git.

## Ethics statement

The studies involving human participants were reviewed and approved by Comité de Ética en la Investigación del Instituto de Neurobiología, Universidad Nacional Autónoma de México. The patients/participants provided their written informed consent to participate in this study.

## Author contributions

EV-C: conceptualization, software, data curation, formal analysis, investigation, methodology, project administration, supervision, validation, visualization, writing—original draft, writing—review, and editing. CI: data curation, investigation, project administration, writing—review, and editing. DM: validation, methodology, writing—original draft, writing—review, and editing. MG: conceptualization, methodology, funding acquisition, project administration, supervision, writing—original draft, writing—review, and editing. All authors contributed to the article and approved the submitted version.

## Funding

This study was supported by grants from DGAPA-PAPIIT (IN 203818) and CONACyT (Fronteras No. 225-2015 and scholarship to EV-C No. 755580).

## Conflict of interest

The authors declare that the research was conducted in the absence of any commercial or financial relationships that could be construed as a potential conflict of interest.

## Publisher’s note

All claims expressed in this article are solely those of the authors and do not necessarily represent those of their affiliated organizations, or those of the publisher, the editors and the reviewers. Any product that may be evaluated in this article, or claim that may be made by its manufacturer, is not guaranteed or endorsed by the publisher.

## Abbreviations

F0: Fundamental frequency
AU: Actions Units
WAIS: Wechsler Adult Intelligence Scale
BANFE: *Batería Neuropsicológica de Funciones Ejecutivas* (Neuropsychological Test of Executive Functions)
ToM: Theory of Mind
RMET: Reading the Mind in the Eyes Test
SST: Short Story Task
AQ: Autism Spectrum Quotient
SSS: Sarcasm Self-Report Scale
