# Supplementary material for: A novel task to evaluate irony comprehension and its essential elements in Spanish speakers
Source: Front Psychol. 2022 Nov 22;13:963666. doi: 10.3389/fpsyg.2022.963666 (PMC9724626; doi:10.3389/fpsyg.2022.963666)
Supplement: Supplementary file 1 [file Data_Sheet_1.ZIP › Supplementary Table 2.pdf]

variable	n	min	max	median	q1	q3	iqr	mad	mean	sd	se	ci
Prosody Irony classification rt	30	0.34	3.0	0.80	0.58	1.34	0.76	0.51	1.00	0.59	0.11	0.22
Prosody Literal classification rt	30	0.35	2.3	0.96	0.68	1.44	0.76	0.54	1.05	0.50	0.09	0.19
Prosody Unrelated classification rt	30	0.25	2.2	0.80	0.61	1.26	0.65	0.36	0.96	0.51	0.09	0.19
FE Irony classification rt	30	0.10	1.5	0.62	0.47	0.75	0.28	0.22	0.65	0.32	0.06	0.12
FE Literal classification rt	30	0.13	1.4	0.59	0.38	0.74	0.36	0.27	0.57	0.28	0.05	0.10
FE Unrelated classification rt	30	0.23	1.4	0.62	0.50	0.86	0.36	0.31	0.70	0.31	0.06	0.12
Prosody Irony score	30	29.00	100.0	86.00	74.50	93.00	18.50	13.34	80.40	18.06	3.30	6.75
Prosody Literal score	30	49.00	99.0	90.50	86.25	94.00	7.75	5.93	88.43	10.29	1.88	3.84
Prosody Unrelated score	30	2.00	98.0	86.00	69.50	93.00	23.50	11.86	78.50	22.17	4.05	8.28
FE Irony score	30	28.00	100.0	91.00	82.00	97.00	15.00	8.90	85.07	18.13	3.31	6.77
FE Literal score	30	47.00	100.0	94.00	85.00	100.00	15.00	8.90	90.57	11.43	2.09	4.27
FE Unrelated score	30	42.00	100.0	95.00	78.50	99.50	21.00	7.41	87.33	15.15	2.77	5.66
RMET	30	10.00	31.0	27.00	25.00	28.00	3.00	2.96	25.27	5.04	0.92	1.88
RMET rt	30	3.45	24.1	8.34	6.99	10.96	3.98	3.67	10.07	5.50	1.00	2.06
AQ	30	7.00	32.0	19.00	15.00	24.25	9.25	6.67	19.37	6.43	1.17	2.40
AQ attention switching	30	2.00	9.0	5.00	3.00	6.75	3.75	2.96	5.03	2.33	0.42	0.87
AQ attention to detail	30	1.00	9.0	6.00	4.00	7.00	3.00	2.96	5.50	2.46	0.45	0.92
AQ communication	30	0.00	7.0	2.00	1.00	3.75	2.75	1.48	2.50	2.24	0.41	0.84
AQ imagination	30	0.00	8.0	3.00	2.00	4.75	2.75	1.48	3.30	2.15	0.39	0.80
AQ social skill	30	0.00	7.0	3.00	1.25	4.00	2.75	2.22	3.03	2.06	0.38	0.77
SSS	30	1.00	7.0	3.44	2.55	4.50	1.95	1.53	3.54	1.56	0.28	0.58
