# Supplementary material for: A novel task to evaluate irony comprehension and its essential elements in Spanish speakers
Source: Front Psychol. 2022 Nov 22;13:963666. doi: 10.3389/fpsyg.2022.963666 (PMC9724626; doi:10.3389/fpsyg.2022.963666)
Supplement: Supplementary file 1 [file Data_Sheet_1.ZIP › Supplementary Table 1.pdf]

variable	min	max	median	q1	q3	iqr	mad	mean	sd	se	ci
Irony context rt	7.0	31.0	12.0	10.0	14.8	4.75	3.71	12.77	4.78	0.872	1.78
Literal context rt	5.0	25.0	11.0	9.0	13.0	4.00	2.96	11.47	3.94	0.719	1.47
Unrelated context rt	6.0	25.0	12.0	9.0	15.0	6.00	4.45	12.30	4.36	0.795	1.63
White lies context rt	6.0	22.0	11.5	9.0	14.0	5.00	3.71	11.90	3.60	0.656	1.34
Irony statement rt	1.0	3.0	1.0	1.0	2.0	1.00	0.00	1.50	0.63	0.115	0.23
Literal statement rt	1.0	2.0	1.0	1.0	2.0	1.00	0.00	1.33	0.48	0.088	0.18
Unrelated statement rt	1.0	3.0	2.0	1.0	2.0	1.00	0.00	1.67	0.55	0.100	0.20
White lies statement rt	1.0	2.0	1.0	1.0	2.0	1.00	0.00	1.37	0.49	0.089	0.18
Irony classification rt	1.9	8.6	2.8	2.4	3.5	1.15	0.73	3.28	1.51	0.275	0.56
Literal classification rt	1.3	3.6	2.1	1.7	2.5	0.75	0.53	2.13	0.57	0.104	0.21
Unrelated classification rt	1.3	4.2	2.4	2.0	3.0	0.93	0.61	2.54	0.75	0.137	0.28
White lies classification rt	1.6	6.0	2.4	2.0	3.3	1.28	0.58	2.78	1.17	0.213	0.43
Irony score	36.0	100.0	93.0	67.8	93.0	25.25	10.38	82.47	19.93	3.639	7.44
Literal score	71.0	100.0	100.0	100.0	100.0	0.00	0.00	96.93	6.93	1.265	2.59
Unrelated score	79.0	100.0	100.0	100.0	100.0	0.00	0.00	97.20	5.99	1.093	2.23
White lies score	50.0	100.0	93.0	86.0	100.0	14.00	10.38	90.30	13.22	2.414	4.94
Raven	5.0	12.0	10.0	9.0	11.0	2.00	1.48	9.63	1.87	0.341	0.70
Verbal fluency	6.0	35.0	21.0	18.0	27.2	9.25	5.19	21.53	6.64	1.212	2.48
SST	5.0	22.0	17.0	14.2	18.0	3.75	2.96	16.03	3.68	0.672	1.38
SST Comprehension	3.0	10.0	9.0	6.2	9.8	3.50	1.48	8.03	2.01	0.367	0.75
SST SMSI	0.0	1.0	0.0	0.0	0.0	0.00	0.00	0.23	0.43	0.079	0.16
SST MSR	2.0	12.0	8.0	7.0	9.0	2.00	1.48	7.77	2.50	0.457	0.93
Block design	5.0	15.0	10.0	7.2	11.0	3.75	2.96	9.47	2.53	0.462	0.94
Block design time	21.0	42.0	28.0	25.0	31.5	6.50	5.19	28.47	5.29	0.966	1.98
Digit span forward	6.0	16.0	10.0	9.0	12.0	3.00	2.96	10.50	2.32	0.423	0.86
Digit span backward	6.0	14.0	8.0	7.0	9.0	2.00	1.48	8.47	1.81	0.331	0.68
