# Supplementary material for: A novel task to evaluate irony comprehension and its essential elements in Spanish speakers
Source: Front Psychol. 2022 Nov 22;13:963666. doi: 10.3389/fpsyg.2022.963666 (PMC9724626; doi:10.3389/fpsyg.2022.963666)
Supplement: Supplementary file 1 [file Data_Sheet_1.ZIP › Supplementary Table 3.pdf]

variable	min	max	median	q1	q3	iqr	mad	mean	sd	se	ci
Context Irony classification rt	1.02	22.9	2.9	2.4	3.90	1.50	1.07	3.76	3.82	0.697	1.43
Context Literal classification rt	1.17	4.9	2.5	2.1	3.35	1.27	0.73	2.73	0.90	0.164	0.34
Context Unrelated classification rt	1.18	4.9	2.6	1.7	3.02	1.27	1.02	2.56	0.96	0.175	0.36
Prosody Irony classification rt	0.97	5.9	2.1	1.6	2.73	1.08	0.87	2.29	1.05	0.192	0.39
Prosody Literal classification rt	0.87	4.7	2.0	1.5	2.47	0.97	0.76	2.06	0.87	0.160	0.33
Prosody Unrelated classification rt	0.95	6.3	1.9	1.6	2.29	0.71	0.56	2.10	1.02	0.187	0.38
FE Irony classification rt	0.74	3.0	1.6	1.2	1.98	0.77	0.57	1.70	0.58	0.105	0.21
FE Literal classification rt	0.82	2.8	1.5	1.3	1.74	0.46	0.36	1.57	0.48	0.088	0.18
FE Unrelated classification rt	0.66	3.5	1.7	1.5	2.46	0.99	0.64	1.97	0.74	0.136	0.28
Context Irony score	0.00	100.0	100.0	92.9	100.00	7.10	0.00	90.96	19.13	3.492	7.14
Context Literal score	57.10	100.0	85.7	78.6	85.70	7.10	10.53	83.73	8.99	1.641	3.36
Context Unrelated score	85.70	100.0	100.0	92.9	100.00	7.10	0.00	97.63	3.89	0.711	1.45
Prosody Irony score	40.50	100.0	88.1	68.5	94.03	25.55	12.31	81.35	16.30	2.977	6.09
Prosody Literal score	65.20	98.6	91.3	85.5	94.20	8.70	6.52	88.47	8.17	1.492	3.05
Prosody Unrelated score	0.00	100.0	82.2	47.6	90.50	42.90	22.91	67.93	31.69	5.785	11.83
FE Irony score	42.10	100.0	81.5	61.2	94.70	33.52	23.50	77.62	19.07	3.481	7.12
FE Literal score	66.70	100.0	93.9	84.8	100.00	15.20	9.04	90.45	10.60	1.936	3.96
FE Unrelated score	5.10	100.0	75.7	46.2	99.35	53.15	36.10	68.13	32.18	5.876	12.02
SST	8.00	25.0	16.5	15.0	21.00	6.00	3.71	17.20	4.03	0.736	1.50
SST Comprehension	4.00	10.0	8.5	7.0	10.00	3.00	2.22	8.13	1.81	0.331	0.68
SST SMSI	0.00	1.0	0.0	0.0	0.75	0.75	0.00	0.27	0.45	0.082	0.17
SST MSR	4.00	15.0	8.5	7.0	11.00	4.00	3.71	8.80	2.94	0.537	1.10
RMET	3.00	33.0	27.0	24.2	28.75	4.50	2.96	25.87	5.25	0.959	1.96
RMET rt	5.77	14.0	8.9	7.2	10.42	3.23	2.45	9.05	2.29	0.419	0.86
AQ	11.00	30.0	20.0	16.0	24.00	8.00	5.93	19.72	4.62	0.858	1.76
AQ attention switching	2.00	9.0	5.0	4.0	7.00	3.00	2.96	5.41	2.03	0.376	0.77
AQ attention to detail	0.00	10.0	5.0	5.0	6.00	1.00	1.48	5.52	1.96	0.363	0.74
AQ communication	0.00	7.0	2.0	1.0	3.00	2.00	1.48	2.17	1.60	0.298	0.61
AQ imagination	0.00	8.0	3.0	2.0	5.00	3.00	1.48	3.31	1.93	0.358	0.73
AQ social skill	1.00	7.0	3.0	2.0	5.00	3.00	1.48	3.31	1.79	0.333	0.68
SSS	1.27	6.5	3.7	2.2	4.33	2.13	1.19	3.48	1.36	0.252	0.52
