# Supplementary figures and images for: A novel task to evaluate irony comprehension and its essential elements in Spanish speakers
Source: Front Psychol. 2022 Nov 22;13:963666. doi: 10.3389/fpsyg.2022.963666 (PMC9724626; doi:10.3389/fpsyg.2022.963666)

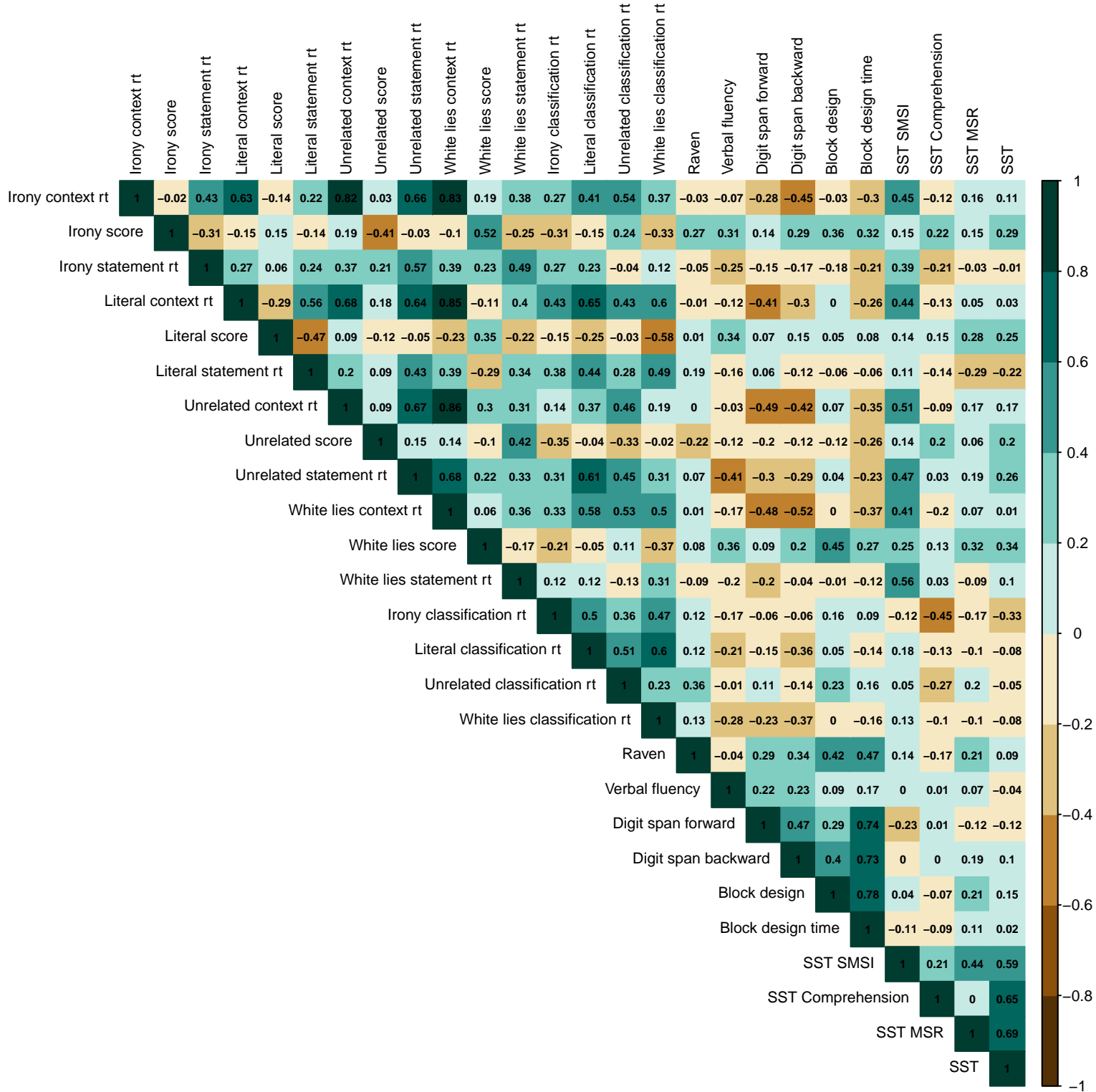

Supplement: Supplementary file 1 [file Data_Sheet_1.ZIP › Supplementary Figure 1.pdf]

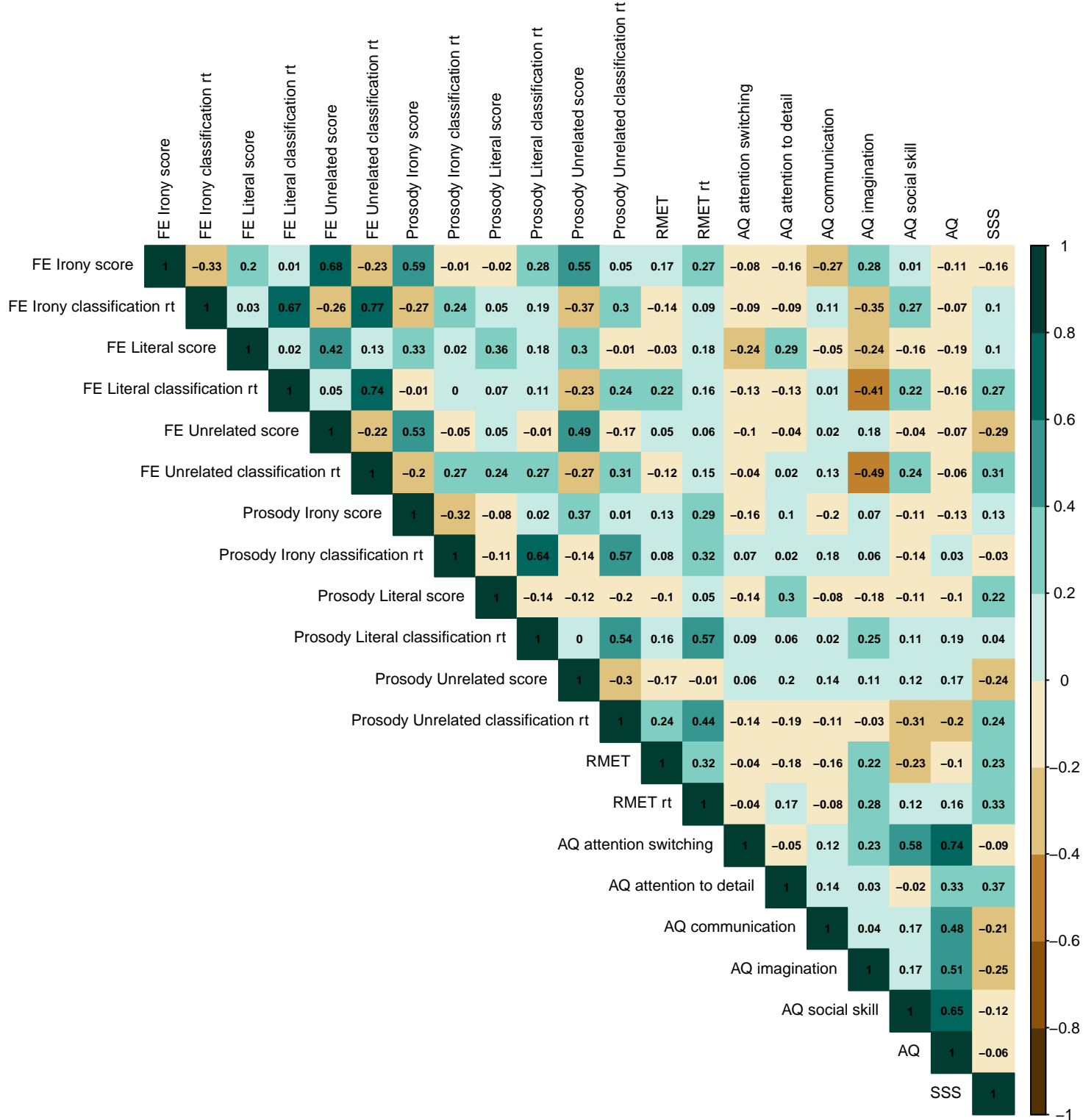

Supplement: Supplementary file 1 [file Data_Sheet_1.ZIP › Supplementary Figure 2.pdf]

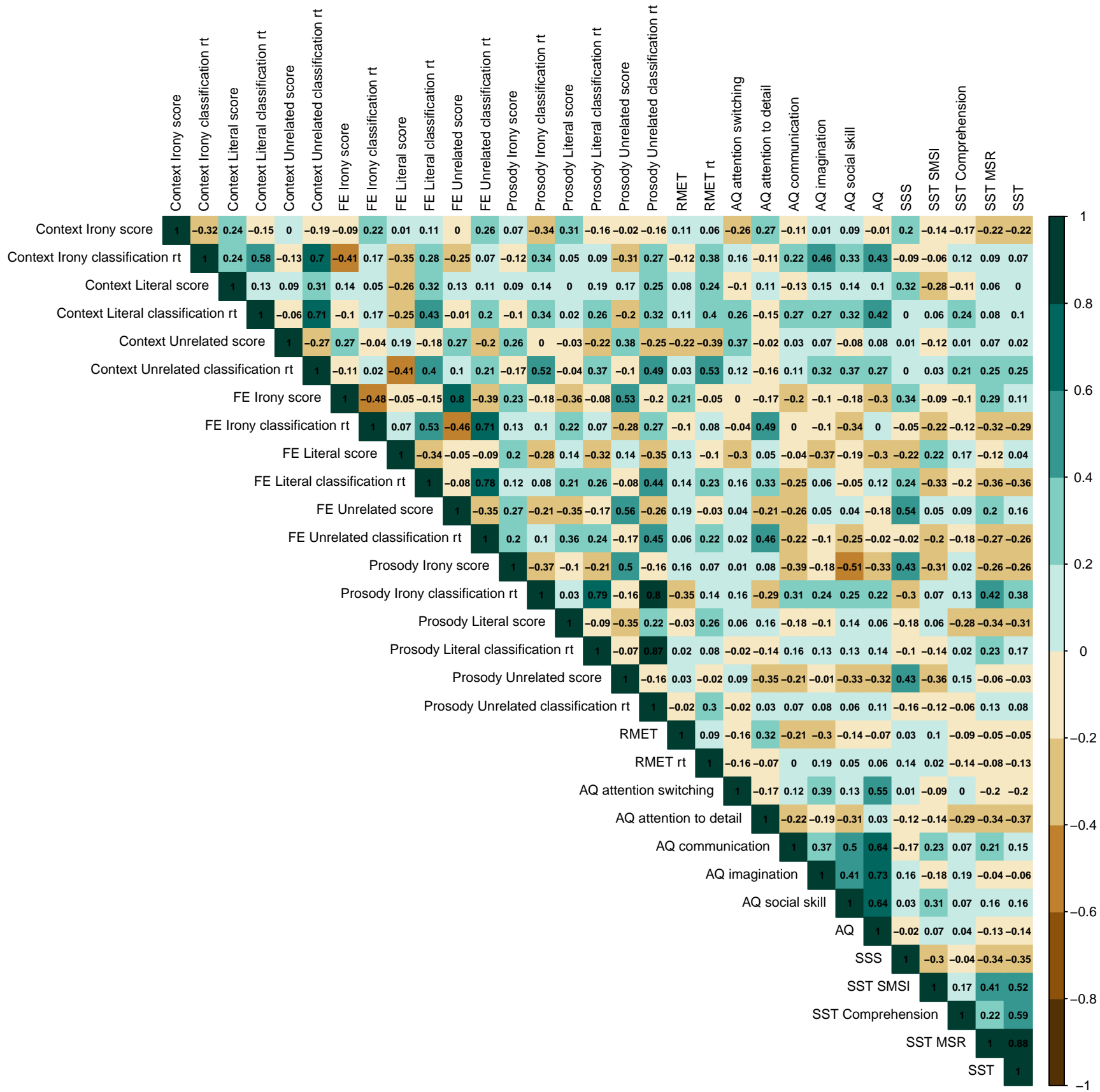

Supplement: Supplementary file 1 [file Data_Sheet_1.ZIP › Supplementary Figure 3.pdf]
